# Agitation Predicts Response of Depression to Botulinum Toxin Treatment in a Randomized Controlled Trial

**DOI:** 10.3389/fpsyt.2014.00036

**Published:** 2014-03-31

**Authors:** M. Axel Wollmer, Nadeem Kalak, Stefanie Jung, Claas de Boer, Michelle Magid, Jason S. Reichenberg, Serge Brand, Edith Holsboer-Trachsler, Tillmann H. C. Kruger

**Affiliations:** ^1^Asklepios Clinic North – Ochsenzoll, Asklepios Campus Hamburg, Medical Faculty, Semmelweis University, Hamburg, Germany; ^2^Psychiatric Clinics of the University of Basel, Basel, Switzerland; ^3^Department of Psychiatry, Social Psychiatry and Psychotherapy, Medical School Hannover, Hannover, Germany; ^4^Department of Psychiatry, University of Texas Southwestern at Seton Family of Hospitals, Austin, TX, USA; ^5^Department of Dermatology, University of Texas Southwestern at Seton Family of Hospitals, Austin, TX, USA

**Keywords:** psychomotor agitation, major depressive disorder, type A botulinum toxins, randomized controlled trial, personalized medicine

## Abstract

In a randomized, controlled trial (*n* = 30), we showed that botulinum toxin injection to the glabellar region produces a marked improvement in the symptoms of major depression. We hypothesized that the mood-lifting effect was mediated by facial feedback mechanisms. Here we assessed if agitation, which may be associated with increased dynamic psychomotor activity of the facial musculature, can predict response to the treatment. To test this hypothesis, we re-analyzed the data of the scales from our previous study on a single item basis and compared the baseline scores in the agitation item (item 9) of the Hamilton Depression Rating Scale (HAM-D) between responders (*n* = 9) and participants who did not attain response (*n* = 6) among the recipients of onabotulinumtoxinA (*n* = 15). Responders had significantly higher item 9 scores at baseline [1.56 + 0.88 vs. 0.33 + 0.52, *t*_(13)_ = 3.04, *d* = 1.7, *p* = 0.01], while no other single item of the HAM-D or the Beck Depression Inventory was associated with treatment response. The agitation score had an overall precision of 78% in predicting response in a receiver operating characteristic (ROC) analysis (area under the curve, AUC = 0.87). These data provide a link between response to botulinum toxin treatment with a psychomotor manifestation of depression and thereby indirect support of the proposed facial feedback mechanism of action. Moreover, it suggests that patients with agitated depression may particularly benefit from botulinum toxin treatment.

## Introduction

Botulinum toxin treatment of the face can impact on mood and affect. After injection of the glabellar region recipients reported an increase in emotional well-being (1) and a reduction in the levels of negative emotions such as fear and sadness (2). Currently, five studies have investigated the clinical use of botulinum toxin injection to the glabellar region in the treatment of depression. All 10 participants in an open case study experienced remission of depression or at least marked reduction in its symptoms (3). Subsequently, in the first randomized, controlled trial (RCT) we showed that a single treatment of the glabellar region with onabotulinumtoxinA may lead to a strong and sustained alleviation of major depression symptoms that had not improved sufficiently through previous antidepressant medication and these findings were confirmed in two other RCTs (4–6). Improvement in the symptoms of depression was also observed in a recent open study (7). Not all patients in our study responded to botulinum toxin treatment (number needed to treat, NNT = 2.13), however, the response rate of 60% in the verum group was high, especially for patients with partly chronic and treatment-resistant depression. With view to future personalized or stratified approaches in the management of depression, it would be helpful to identify predictors of response to botulinum toxin treatment (8). We conducted our study based upon the facial feedback hypothesis, which implies that negative emotions associated with depression are maintained and reinforced by proprioceptive afferences from the facial muscles that express these emotions. The interruption of this feedback loop by the paralysis of the respective muscles may accomplish the mood-lifting effect of botulinum toxin. Therefore, it seems likely that the intervention may be particularly effective in depressed patients with a high baseline activity of the targeted muscles. Facial features like frown lines or specifically the “omega melancholicum” and Veraguth’s folds are possible clinical manifestations of this activity and are associated with agitation, i.e., a generalized increase in dynamic psychomotor activity in depression (9).

To test the hypothesis that increased psychomotor activity in general and in the glabellar region of the face in particular may predict response of depression to botulinum toxin treatment, we investigated, whether baseline agitation and frown line severity were associated with treatment outcome measures in our trial.

## Materials and Methods

The original RCT (ClinicalTrials.gov, NCT00934687) was conducted at two centers in Switzerland and Germany as previously described [open access at http://www.sciencedirect.com/science/article/pii/S0022395612000386; Ref. (4)]. It had the approval of the local ethic committees and the regulatory authorities. In brief, 30 eligible, predominantly female outpatients with an average age of about 50 years with moderate and partly chronic and treatment-resistant unipolar depression and presence of frown lines gave informed consent to receive adjunctive injections of either onabotulinumtoxinA (Vistabel^®^, Botox^®^ Cosmetic, Allergan) or a saline placebo to the glabellar region. Participants were assessed using the German versions of the Structured Interview Guide for the Hamilton Depression Rating Scale with Atypical Depression Supplement (SIGH-ADS) (10), the Beck Depression Inventory (BDI) self-rating questionnaire, the Clinical Global Impressions Scale (CGI), and a four-point Clinical Severity Score for Glabellar Frown Lines (CSS-GFL) (11). Treatment response was defined as a ≥50% reduction after 6 weeks, which was the primary end point of the trial.

Here, we re-assessed our data in a single item analysis of the depression scales: in the verum group and separately in the placebo group, responders and those who did not fulfill the response criteria were compared for the collected baseline characteristics including single item scores of the HAM-D scale and the BDI applying Student’s *t*-tests or Welch-test for continuous, and Fisher’s exact tests for categorical variables. All tests were two-sided and variance is reported as standard deviation. Effect sizes are reported as Cohen’s *d*. A receiver operating characteristic (ROC) curve analysis was performed to estimate the sensitivity, specificity, and overall precision of the baseline HAM-D agitation item (item 9) score (0 = “none,” 1 = “fidgetiness,” 2 = “playing with hands, hair, etc.,” 3 = “moving about, cannot sit still,” and 4 = “hand wringing, nail biting, hair pulling, biting of lips”) in predicting treatment response. According to the determined cut-off, participants were classified into a lower agitation (≤1, LA) and a higher agitation (≥2, HA) group. For a more precise assessment of glabellar frown line severity, we introduced intermediate steps to the CSS-GFL and ranked all 30 participants of the study according to their individual frown line severity at maximum frowning (1 = strongest, 30 = weakest frown line). Test results with unadjusted *p*-values of ≤0.05 were considered significant. Statistical analyses were conducted using SPSS 19.0 for Windows.

## Results

**Figure 1:**
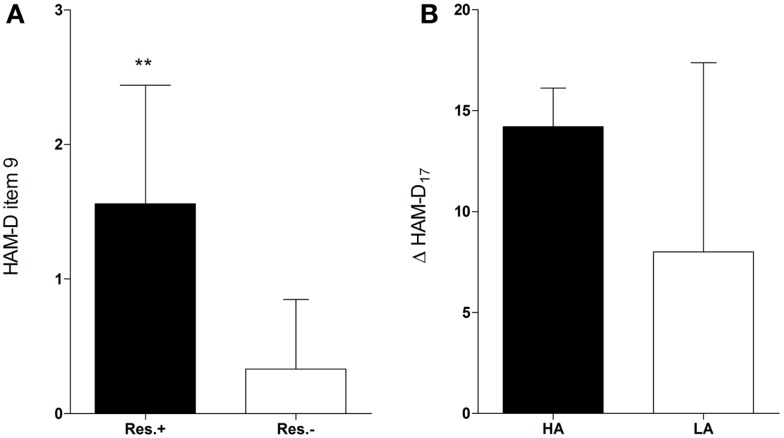
**Higher baseline agitation in responders**. Responders (Res.±, *n* = 9) had significantly higher baseline scores in the agitation item (item 9) of the HAM-D than participants who did not fulfill the response criterion [Res.−, *n* = 6; 1.56 ± 0.88 vs. 0.33 ± 0.52, *t*_(13)_ = 3.04, *d* = 1.7, *p* = 0.01** **(A)**]. Participants with higher agitation scores at baseline (HA) tended to have a greater improvement in the HAM-D_17_ score (Δ HAM-D_17_) compared to those with lower agitation scores [LA, *n* = 10; 14.2 ± 1.92 vs. 8.0 ± 9.37, *w*_(13)_ = 2.01, *d* = 0.92, *p* = 0.07 **(B)**].

The participants who received onabotulinumtoxinA treatment (*n* = 15) and were responders (*n* = 9) had significantly higher baseline scores in the agitation item of the HAM-D scale than participants who did not fulfill the response criterion [*n* = 6; 1.56 ± 0.88 vs. 0.33 ± 0.52, *t*_(13)_ = 3.04, *d* = 1.7, *p* = 0.01, Figure 1A]. Response was not associated with baseline scores of any other HAM-D item. Hence, in the BDI, which does not assess agitation, no baseline score of any item was associated with response (Table S1 in Supplementary Material). Baseline agitation scores did not differ significantly between the verum and the placebo group [1.07 ± 0.96 vs. 0.87 ± 1.06, *t*_(28)_ = 0.54, *d* = 0.2, *p* = 0.59] and were not associated with response in the placebo group [1.0 ± 1.41 vs. 0.85 ± 1.07, *t*_(13)_ = 0.18, *d* = 0.2, *p* = 0.86].

**Figure 2:**
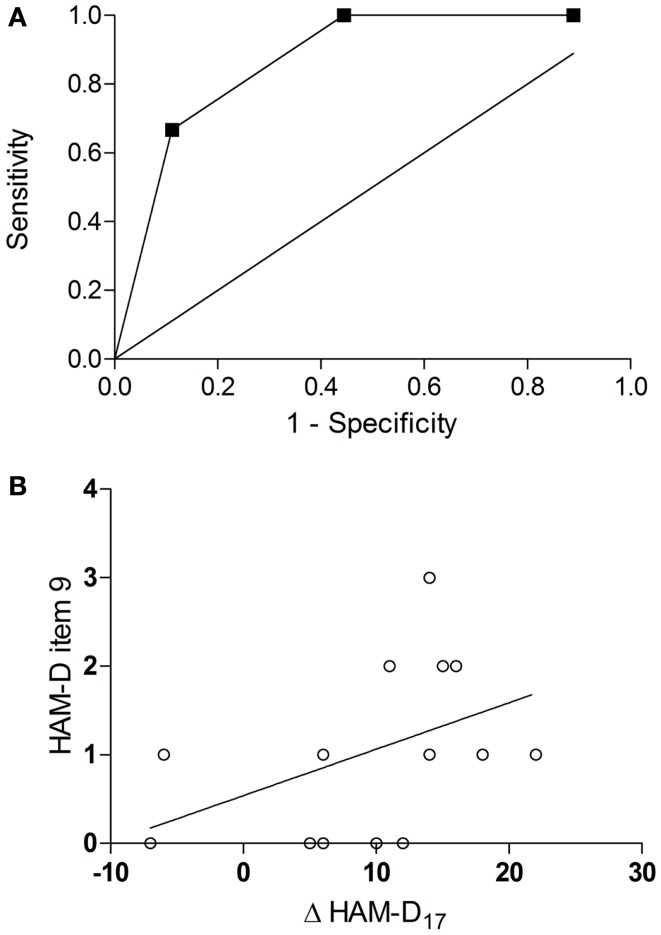
**Prediction of response by baseline agitation**. ROC curve for the baseline scores in the agitation item of the HAM-D as a predictor of response [AUC = 0.87, 95% CI = 0.69–1.05, *p* = 0.02 **(A)**] correlation of baseline agitation scores with improvement in the HAM-D_17_ total score (Δ HAM-D_17_) from baseline to the visit after 6 weeks [*r* = 0.45, *p* = 0.10**(B)**].

An ROC curve analysis revealed that the baseline agitation score was a good predictor of response (Figure 2A): the area under the curve (AUC) was 0.87 (95% CI = 0.69–1.05, *p* = 0.02). With a sensitivity of 100% (1.0, 95% CI = 0.54–1.0) and a specificity of 56% (0.56, 95% CI = 0.21–0.86), a cut-off between 1 and 2 (1.5) achieved the best overall precision (78%). Accordingly, all patients in the verum group with a baseline agitation score of ≥2 (HA, *n* = 5) but only a minority of those with a baseline agitation score of ≤1 (LA, *n* = 10) attained response (100 vs. 40%, risk ratio = 2.5, 95% CI = 1.17–5.34, *p* = 0.04, Table 1). HA participants tended to have a greater improvement in the HAM-D_17_ total score from baseline to the visit after 6 weeks (Δ HAM-D_17_) compared to LA participants [14.2 ± 1.92 vs. 8.0 ± 9.37, *w*_(13)_ = 2.01, *d* = 0.92, *p* = 0.07, Figure 1B] and there was a trend toward a correlation of baseline agitation scores with Δ HAM-D_17_ (*r* = 0.45, *p* = 0.10, Figure 2B).

**Table 1:** **Association of agitation with response**.

	Agitation	
Response	HA (%)	LA (%)
+	5 (100)	4 (40)
−	0 (0)	6 (60)

*Fisher’s exact test *p* = 0.04, risk ratio = 2.5, 95% CI = 1.17–5.34*.

*Distribution of the patients of the verum group according to baseline agitation (HA, LA) and response. All HA participants are responders, indicating a positive predictive value of higher agitation at baseline for response to onabotulinumtoxinA treatment. Conversely, lower agitation at baseline does not preclude response*.

There was only a non-significant reduction in the agitation score from baseline to the visit after 6 weeks (Δ HAM-D-9) in the verum group [from 1.07 ± 0.96 to 0.67 ± 1.11, *t*_(14)_ = 1.57, *p* = 0.14], which was driven by the HA sub-group [from 2.2 ± 0.45 to 1.2 ± 1.11, *t*_(4)_ = 2.24, *p* = 0.09].

Baseline severity of glabellar frown lines as a possible facial correlate of psychomotor activity was not associated with response. Neither the original CSS-GFL [2.22 ± 0.44 vs. 2.17 ± 0.41, *t*_(13)_ = 0.254, *d* = 0.12, *p* = 0.81] nor the modified version with the newly introduced intermediate steps [2.22 ± 0.51 vs. 2.0 ± 0.77, *t*_(13)_ = 0.68, *d* = 0.34, *p* = 0.51] nor the average rankings [15.67 ± 7.09 vs. 21.33 ± 9.73, *t*_(13)_ = 1.31, *d* = 0.66, *p* = 0.21] differed between responders and those who did not respond in the verum group. The change in the CSS-GFL between baseline and the visit after 6 weeks (Δ CSS-GFL) was similar between the two groups [0.89 ± 0.33 vs. 0.83 ± 0.41, *t*_(13)_ = 0.29, *d* = 0.16, *p* = 0.78]. However, we found a correlation of the baseline score in the modified version of the CSS-GFL and of the baseline rank of frown line severity with improvement in the HAM-D_17_ total score from baseline to the visit after 16 weeks (*r* = 0.51, *p* = 0.05; *r* = −0.68, *p* = 0.01). These correlations were not significant at the primary end point after 6 weeks (*r* = 0.31, *p* = 0.26; *r* = −0.46, *p* = 0.08). Glabellar frown line severity was not associated with agitation (*p* ≥ 0.08).

## Discussion

Using an RCT design, we previously demonstrated that treatment of the glabellar region with onabotulinumtoxinA can reduce the symptoms of major depression (4).

The mechanisms underlying this clinical effect are unknown. However, a facial feedback mechanism is supported by other studies that show effects of botulinum toxin treatment on emotional perception (12–14), as well as by experiments in which facial muscle activity was influenced by other means (15–17). Accordingly, we hypothesized that clinical improvement in depression would most likely be mediated by a modification of direct proprioceptive feedback induced by a reduction of primarily increased facial muscle activity. Thus, patients with high base levels of glabellar muscle activity may benefit more from the treatment than patients with low base levels of such activity. High activation levels may manifest in facial features like frown lines and specifically the “omega melancholicum,” which is associated with psychomotor agitation in depression (9). In support of our hypothesis, we showed that responders to botulinum toxin treatment had higher agitation levels at baseline than participants who did not respond to the treatment and that a higher baseline level of agitation, as measured by the item 9 of the HAM-D scale (>1), had a good positive predictive value referring to response to botulinum toxin treatment in our study (18). However, a lower baseline level of agitation (≤1) did not preclude response. These findings were specific to agitation as no other single item was associated with response. Agitation was associated with response only in the verum but not in the placebo group and, therefore, not a general indicator of a better outcome in our sample. Association of psychomotor symptoms with treatment outcome has been observed before; however, these observations were inconsistent (19). Floor effects in the LA participants may explain the non-significant Δ HAM-D-9. Some reduction in the item 9 score after treatment was conferred by the HA patients. This suggests that agitation was probably rather a psychomotor state marker of depression than a psychomotor trait of the respective individuals.

The described association of agitation with response is in line with facial feedback as a possible mechanism underlying the observed clinical improvement, because it links the improvement to a psychomotor endophenotype of depression that may comprise facial muscle activation patterns targeted by the treatment. At the same time, the retrospective identification of a predictor for response within the verum group that is mechanistically plausible may be regarded as an argument against a major role of placebo effects.

We found only a weak link between glabellar frown line severity at baseline with clinical improvement in depression. Although frown line severity may not necessarily directly reflect the actual facial muscle activity, it is probably influenced by an individual’s facial psychomotor history. Thus, this link may also support a facial feedback mechanism of action. The ability to produce a moderate frown line at maximum frowning was an inclusion criterion in our study (4). This may have led to a reduction in the variability of frown line severity in our sample and to an underestimation of its correlation with improvement in depression. In another RCT, frown line severity was not an inclusion criterion and in this trial there was a trend toward association of reduction in frown line severity and improvement in depression (5). The “omega melancholicum” is associated with agitation in depression (9). However, we did not observe association of frown line severity with agitation in the participants of our study. In future studies, a more thorough and more direct assessment of facial muscle activity should be applied: facial electromyography and standardized video recordings, which can be evaluated using, e.g., the facial action coding system (FACS) (20) may be used to investigate if facial psychomotor features can predict response of depression to botulinum toxin treatment and customize its dosage and injection sites.

A limitation of the present study is the rather low number (*n* = 15) of participants. Thus it cannot be excluded, that the observed association of agitation with response is a statistical type I error. Driven by the hypothesis that a psychomotor phenotype with increased activity may show a better response, we investigated primarily the association of response and agitation and frown line severity. We acknowledge that a higher response rate in participants with higher agitation levels at baseline was not a stated prediction of the original study. Thus, the association of agitation with response would not remain significant, if we applied a Bonferroni correction for the multiple comparisons we did when we looked at the other single items of the HAM-D and the BDI scales and the other baseline variables to assess the specificity of the observed association. Therefore, our observation needs to be confirmed in further and larger studies. In the meantime, we had the opportunity to verify our finding in another recent RCT on botulinum toxin in the treatment of depression that used the HAM-D scale (6). In this trial, only one of the 28 recipients of botulinum toxin injections had an HA score at the baseline of the treatment period and she turned out to be a responder. One may speculate that the relative lack of HA subjects could partly explain the slightly lower overall response rate in this study.

The identification of agitation as a predictor of response may be helpful in the development of stratified medicine approaches in the treatment of depression. Notably, our patients did not have overt agitated depression but only varied in their agitation scores in the HAM-D scale. Hence, none of the participants scored 4 in the agitation item. It is possible that patients with clinical agitated depression may respond particularly well to botulinum toxin treatment, which corresponds to our personal clinical experience. Moreover, our findings suggest that glabellar injection of botulinum toxin may also be effective as a treatment of other psychiatric disorders associated with negative emotions and increased psychomotor activity.

## Author Contributions

M. Axel Wollmer was the principal investigator in Basel, Switzerland. He conceived the hypothesis, wrote the manuscript, and calculated statistics. Nadeem Kalak computed study data and calculated statistics. Stefanie Jung helped with statistics and prepared tables. Claas de Boer computed study data. Michelle Magid and Jason S. Reichenberg contributed data to the discussion of the manuscript. Serge Brand advised and supervised with statistical analyses. Edith Holsboer-Trachsler contributed to the design of the original study and to the discussion of the paper. Tillmann H. C. Kruger was the principal investigator in Hannover, Germany. He contributed to the writing of the manuscript and prepared all figures. All authors reviewed the manuscript and have given approval of its publication.

## Conflict of Interest Statement

M. Axel Wollmer received honoraria for talks from Merz, Eli Lilly, and Novartis. Edith Holsboer-Trachsler received research grants from Actelion, Cephalon, Eli Lilly, Essex/MSD, Servier, and Vifor, received compensation for CME activity from AstraZeneca, Bristol-Myers Squibb, Eli Lilly, Essex/MSD, Permamed, Pfizer, Servier, and Vifor, and is a member of the advisory boards of Eli Lilly, Lundbeck, Pfizer, and Servier. Tillmann H. C. Kruger received honoraria for talks from Boehringer, Servier, Pfizer, and Ferring. These activities were all unrelated to the study. In April 2012, i.e., after conclusion and publication of the study, M. Axel Wollmer and Tillmann H. C. Kruger became members of the advisory board of Allergan. In November 2012, Michelle Magid became a consultant with Allergan. Nadeem Kalak, Stefanie Jung, Claas de Boer, Jason S. Reichenberg, and Serge Brand declare no conflict of interest.

## Supplementary Material

The Supplementary Material for this article can be found online at http://www.frontiersin.org/Journal/10.3389/fpsyt.2014.00036/abstract
