# Supplementary material for: Agitation Predicts Response of Depression to Botulinum Toxin Treatment in a Randomized Controlled Trial
Source: Front Psychiatry. 2014 Mar 31;5:36. doi: 10.3389/fpsyt.2014.00036 (PMC3978251; doi:10.3389/fpsyt.2014.00036)
Supplement: Supplementary file 1 [file Data_Sheet1.DOC]

SUPPLEMENTARY TABLE

**Wollmer et al.. Agitation predicts response of depression to botulinum toxin treatment in a randomized controlled trial**

| **Supplementary table 1** Baseline characteristics of verum group, response attained (Res.+, >50% reduction in HAM-D_17_ score six weeks after baseline) vs. response not attained (Res.-, <50% reduction in HAM-D_17_ score six weeks after baseline) | | | | | | | |
| --- | --- | --- | --- | --- | --- | --- | --- |
| ***Variable*** | **Res.+ (n=9)** | | **Res.- (n=6)** | | **test** |  | **significance** |
|  | | | | | | | |
| ***Demographic data*** | **n** | **%** | **n** | **%** |  |  |  |
| Women | 9 | 100 | 3 | 50 | Fisher’s exact |  | **p=0.04** |
| Living alone | 2 | 22.2 | 3 | 50 | Fisher’s exact |  | p=0.33 |
| Centre Basel | 4 | 44.4 | 2 | 33.3 | Fisher’s exact |  | p=1.00 |
|  | **mean** | **SD** | **mean** | **SD** |  | **df** |  |
| Age. years | 52.00 | 8.02 | 52.50 | 7.50 | t=0.12 | 13 | p=0.91 |
| Education. years | 14.22 | 2.91 | 13.83 | 1.60 | t=-0.30 | 13 | p=0.77 |
| BMI | 24.98 | 4.01 | 29.62 | 11.01 | t=1.17 | 13 | p=0.26 |
|  | | | | | | | |
| ***Specification of depression*** | **n** | **%** | **n** | **%** |  |  |  |
| Recurrent F33.xx | 7 | 77.8 | 4 | 66.7 | Fisher’s exact |  | p=1.00 |
| Moderate F3x.1x | 7 | 77.8 | 4 | 66.7 | Fisher’s exact |  | p=1.00 |
| Somatic syndrome F3x.x1 | 6 | 66.7 | 3 | 50 | Fisher’s exact |  | p=0.62 |
| History of dysthymic disorder | 2 | 22.2 | 1 | 16.7 | Fisher’s exact |  | p=1.00 |
| Acute | 4 | 44.4 | 2 | 33.3 | Fisher’s exact |  | p=1.00 |
| Chronic IE | 3 | 33.3 | 3 | 50 | Fisher’s exact |  | p=0.62 |
|  | **mean** | **SD** | **mean** | **SD** |  | **df** |  |
| Duration of MDD. years | 17.78 | 13.55 | 14.33 | 9.93 | t=0.53 | 13 | p=0.60 |
| Duration of IE. months | 20.67 | 22.73 | 35.00 | 36.27 | t=0.95 | 13 | p=0.36 |
| Number of episodes | 6.44 | 4.45 | 13.00 | 18.95 | w=0.83 | 13 | p=0.44 |
| HAM-D_17_ | 22.00 | 3.84 | 20.50 | 5.17 | t=0.65 | 13 | p=0.53 |
| HAM-D_21_ | 23.78 | 3.77 | 22.50 | 4.14 | t=0.62 | 13 | p=0.55 |
| ADS | 8.78 | 2.99 | 9.17 | 6.55 | t=0.16 | 13 | p=0.88 |
| SIGH-ADS | 30.78 | 6.16 | 29.67 | 10.46 | t=0.26 | 13 | p=0.80 |
| BDI | 25.67 | 8.70 | 29.00 | 9.65 | t=0.70 | 13 | p=0.50 |
| CGI | 4.56 | 0.53 | 4.83 | 0.75 | t=0.85 | 13 | p=0.41 |
| ***Antidepressant medication*** | **n** | **%** | **n** | **%** |  |  |  |
| SSRI | 5 | 55.6 | 1 | 16.7 | Fisher’s exact |  | p=0.29 |
| SNRI | 3 | 33.3 | 1 | 16.7 | Fisher’s exact |  | p=0.60 |
| Mirtazapine | 1 | 11.1 | 0 | 0 | Fisher’s exact |  | p=1.00 |
| Tricyclic compounds | 1 | 11.1 | 3 | 50 | Fisher’s exact |  | p=0.24 |
| Other compounds | 0 | 0 | 2 | 33.3 | Fisher’s exact |  | p=0.14 |
| Combination | 2 | 22.2 | 1 | 16.7 | Fisher’s exact |  | p=1.00 |
|  | **mean** | **SD** | **mean** | **SD** |  | **df** |  |
| Treatment trials during IE | 1.56 | 1.01 | 1.17 | 0.41 | t=0.88 | 13 | p=0.39 |
| Treatment resistance stage | 1.00 | 0.87 | 1.00 | 0.00 | w=0.00 | 13 | p=1.00 |
| Current antidepressants | 1.11 | 0.60 | 1.17 | 0.41 | t=0.20 | 13 | p=0.85 |
| Adequate dose | 1.11 | 0.60 | 0.83 | 0.41 | t=0.99 | 13 | p=0.34 |
| Stable treatment. months | 16.00 | 17.18 | 21.75 | 14.30 | t=0.68 | 13 | p=0.51 |
| ***Further characteristics*** | | | | | | | |
| CSS-GFL | 2.22 | 0.44 | 2.17 | 0.41 | t=0.25 | 13 | p=0.81 |
| SCID II | 3.38 | 2.77 | 4.17 | 1.94 | t=0.60 | 12 | p=0.56 |
| CEQ I 1 | 7.00 | 2.29 | 5.33 | 2.73 | t=1.28 | 13 | p=0.22 |
| CEQ I 2 | 5.67 | 1.80 | 5.00 | 2.61 | t=0.59 | 13 | p=0.57 |
| CEQ I 3 | 6.22 | 2.11 | 5.83 | 3.76 | w=0.23 | 13 | p=0.82 |
| CEQ I 4 | 52.22 | 18.56 | 50.00 | 20.98 | t=0.22 | 13 | p=0.83 |
| CEQ II 1 | 5.67 | 1.94 | 5.50 | 2.95 | t=0.13 | 13 | p=0.90 |
| CEQ II 2 | 55.56 | 25.06 | 36.67 | 23.38 | t=1.47 | 13 | p=0.17 |
| Appraisal of cosmetic change | 2.44 | 0.88 | 2.67 | 0.52 | t=0.55 | 13 | p=0.59 |
| ***HAM-D21single items*** |  |  |  |  |  |  |  |
| H1 Depressed mood | 2.89 | 1.05 | 3.00 | 0.63 | t=0.23 | 13 | p=0.82 |
| H2 Feelings of guilt | 1.44 | 1.13 | 1.33 | 0.52 | w=0.26 | 13 | p=0.80 |
| H3 Suicide | 0.33 | 0.71 | 1.17 | 1.17 | t=1.73 | 13 | p=0.11 |
| H4 Insomnia early | 1.00 | 0.87 | 0.83 | 0.98 | t=0.35 | 13 | p=0.74 |
| H5 Insomnia middle | 1.44 | 0.88 | 1.33 | 0.82 | t=0.25 | 13 | p=0.81 |
| H6 Insomnia late | 0.78 | 0.97 | 1.00 | 0.89 | t=0.45 | 13 | p=0.66 |
| H7 Work and activities | 2.44 | 0.73 | 2.67 | 0.52 | t=0.65 | 13 | p=0.53 |
| H8 Psychomotor retardation | 1.11 | 0.60 | 0.67 | 0.52 | t=1.48 | 13 | p=0.16 |
| H9 Agitation | 1.56 | 0.88 | 0.33 | 0.52 | t=3.04 | 13 | **p=0.01** |
| H10 Anxiety (psychological) | 1.89 | 1.17 | 1.17 | 1.47 | t=1.06 | 13 | p=0.31 |
| H11 Anxiety (somatic) | 1.89 | 1.05 | 2.83 | 0.98 | t=1.74 | 13 | p=0.11 |
| H12 Somatic symptoms (gastrointestinal) | 0.78 | 0.44 | 0.50 | 0.55 | t=1.09 | 13 | p=0.30 |
| H13 Somatic symptoms general | 1.44 | 0.73 | 1.00 | 0.63 | t=1.22 | 13 | p=0.25 |
| H14 Genital symptoms | 2.00 | 0.00 | 2.00 | 0.00 | NaN |  | NaN |
| H15 Hypochondria | 0.44 | 0.53 | 0.67 | 0.82 | t=0.65 | 13 | p=0.53 |
| H16 Loss of weight | 0.33 | 0.71 | 0.00 | 0.00 | w=1.41 | 13 | p=0.20 |
| H17 Insight | 0.22 | 0.67 | 0.00 | 0.00 | t=0.81 | 13 | p=0.44 |
| H18 Diurnal variation | 1.11 | 0.78 | 0.83 | 0.98 | t=0.61 | 13 | p=0.55 |
| H19 Depersonalization and derealization | 0.22 | 0.67 | 0.33 | 0.82 | t=0.29 | 13 | p=0.78 |
| H20 Paranoid symptoms | 0.22 | 0.44 | 0.33 | 0.52 | t=0.45 | 13 | p=0.66 |
| H21 Obsessional and compulsive symptoms | 0.22 | 0.44 | 0.50 | 0.84 | t=0.85 | 13 | p=0.41 |
| ***ADS single items*** |  |  |  |  |  |  |  |
| A1 Social withdrawal | 2.89 | 0.78 | 2.33 | 0.82 | t=1.33 | 13 | p=0.21 |
| A2 Weight gain | 0.33 | 0.71 | 0.33 | 0.82 | t=0.00 | 13 | p=1.00 |
| A3 Increase of appetite | 0.33 | 0.71 | 0.50 | 1.22 | t=0.34 | 13 | p=0.74 |
| A4 Increase of food intake | 0.33 | 0.71 | 0.50 | 1.22 | t=0.34 | 13 | p=0.74 |
| A5 Craving for carbohydrates | 0.33 | 0.50 | 1.00 | 1.10 | t=1.61 | 13 | p=0.13 |
| A6 Hypersomnia | 0.89 | 1.05 | 0.50 | 0.84 | w=0.79 | 13 | p=0.44 |
| A7 Tiredness | 2.44 | 1.33 | 2.83 | 1.17 | t=0.58 | 13 | p=0.57 |
| A8 Variation Type B | 1.22 | 0.83 | 1.17 | 1.47 | t=0.09 | 13 | p=0.93 |
| ***BDI single items*** |  |  |  |  |  |  |  |
| A Sadness | 1.11 | 0.78 | 1.67 | 1.03 | t=1.19 | 13 | p=0.26 |
| B Pessimism/Hopelessness | 1.44 | 1.42 | 1.83 | 0.98 | w=0.63 | 13 | p=0.54 |
| C Feelings of failure | 1.22 | 1.09 | 1.67 | 1.21 | t=0.74 | 13 | p=0.47 |
| D Loss of pleasure | 1.56 | 0.88 | 2.17 | 0.75 | t=1.39 | 13 | p=0.19 |
| E Guilty feelings | 1.44 | 1.01 | 1.83 | 1.17 | t=0.69 | 13 | p=0.51 |
| F Punishment feelings | 0.78 | 1.30 | 1.33 | 1.37 | t=0.79 | 13 | p=0.44 |
| G Self-dislike | 1.22 | 0.97 | 1.17 | 1.33 | t=0.09 | 13 | p=0.93 |
| H Self-criticalness | 1.44 | 1.24 | 2.17 | 0.98 | t=1.20 | 13 | p=0.25 |
| I Suicidal thoughts | 0.56 | 0.53 | 0.83 | 0.75 | t=0.85 | 13 | p=0.41 |
| J Crying | 1.22 | 1.09 | 1.50 | 1.22 | t=0.46 | 13 | p=0.65 |
| K Irritability | 1.67 | 1.00 | 0.83 | 0.75 | t=1.73 | 13 | p=0.11 |
| L Loss of interest | 1.33 | 0.71 | 1.33 | 1.21 | t=0.00 | 13 | p=1.00 |
| M Indecisiveness | 2.11 | 0.60 | 2.33 | 0.52 | t=0.74 | 13 | p=0.47 |
| N Appearance | 1.11 | 0.93 | 1.00 | 1.55 | w=0.16 | 13 | p=0.88 |
| O Loss of energy | 1.11 | 0.78 | 1.67 | 0.82 | t=1.33 | 13 | p=0.21 |
| P Sleep pattern | 1.33 | 9.71 | 0.67 | 0.52 | t=1.98 | 13 | p=0.07 |
| Q Tiredness or fatigue | 1.44 | 0.53 | 1.83 | 0.75 | t=1.18 | 13 | p=0.26 |
| R Loss of appetite | 0.89 | 0.60 | 0.33 | 0.82 | t=-1.52 | 13 | p=0.15 |
| S Loss of weight | 0.22 | 0.44 | 0.00 | 0.00 | w=1.51 | 13 | p=0.17 |
| T Hypochondria | 0.33 | 0.71 | 0.67 | 0.82 | t=0.84 | 13 | p=0.42 |
| U Loss of libido | 2.11 | 0.78 | 2.17 | 0.98 | t=0.12 | 13 | p=0.91 |
| Abbreviations and statistics are used according to Table 1 and supplemental table 2 of *Wollmer et al., 2012* (open access at <http://www.sciencedirect.com/science/article/pii/S0022395612000386>; 4). As already reported for the whole sample (verum and placebo group, n=30) female sex was associated with response (**p=0.04**). No other demographic or clinical baseline variable was associated with response. In the single item analyses of the depression scales only the agitation item (item 9) of the HAM-D scale was associated with response (**p=0.01**). | | | | | | | |
